# Adherence to a standardized infection reduction bundle decreases surgical site infections after colon surgery: a retrospective cohort study on 526 patients

**DOI:** 10.1186/s13037-021-00285-7

**Published:** 2021-04-08

**Authors:** Marlon A. Guerrero, Brandie Anderson, Gordon Carr, Kara L. Snyder, Patrick Boyle, Sharon A. Ugwu, Melissa Davis, Susan K. Bohnenkamp, Valentine Nfonsam, Taylor S. Riall

**Affiliations:** 1 University of Arizona, Surgery, Arizona Tucson, USA; 2 Banner University Medical Center, Tucson, Arizona USA

## Abstract

**Background:**

Colon surgical site infections (SSI) are detrimental to patient safety and wellbeing. To achieve clinical excellence, our hospital set to improve patient safety for those undergoing colon surgery. Our goal was to implement a perioperative SSI prevention bundle for all colon surgeries to reduce colon surgery SSI rates.

**Methods:**

This retrospective cohort study evaluated the impact of implementing a perioperative SSI prevention bundle in patients undergoing colon surgery at Banner University Medical Center - Tucson. We compared SSI rates between the Pre- (1/1/2016 to 12/31/2016) and post-bundle (1/1/2017 to 12/31/2017) cohorts using a chi-square test.

**Results:**

In total, we included 526 consecutive patients undergoing colon surgery in our study cohort; 277 pre-bundle and 249 post-bundle implementation. The unadjusted SSI rates were 8.7 % and 1.2 %, pre- and post-bundle, respectively. Our CMS reportable standard infection rate decreased by 85.4 % from 3.08 to 0.45 after implementing our SSI prevention bundle.

**Conclusions:**

Implementing a standardized colon SSI prevention bundle reduces the overall 30-day colon SSI rates and national standardized infection rates. We recommend implementing colon SSI reduction bundles to optimize patient safety and minimize colon surgical site infections.

## Background

Colon surgery is associated with a high risk of surgical site infections (SSIs), with reported rates up to 18 % [1–3]. The consequences of SSIs after colon surgery are significant. Not only do SSIs prolong hospital length of stay and hospital costs [3], but they are also associated with an increased risk of death [1]. It has been shown that implementing colon bundles effectively reduce SSIs, however, such bundles are still not universally employed and there are varying elements included in the different bundles [4–6].

At our institution, colon SSIs exceeded national targets and were negatively affecting our publicly reported hospital performance. As a result, we launched a quality improvement initiative to improve the care of our patients by reducing colon SSIs. In this study, we report the quality improvement process and results of our colon SSI reduction bundle (Colon Bundle).

## Methods

### Study design

This retrospective cohort study included patients undergoing colon surgeries at Banner University Medical Center-Tucson, a 487-bed acute-care, level 1 Trauma Center. Our project was a quality improvement program focusing on standardizing our practice with current national standards and therefore did not meet the criteria to be considered a Human Subject Research, and it was deemed exempt from an IRB review.

### Inclusion and exclusion criteria

We included all patients undergoing elective and emergent colon operations between January 1, 2016, and December 31, 2017, in our study. Patients operated consecutively between January 1, 2016, and December 31, 2016, represented our control group. The Colon Bundle initiative began on January 1, 2017. All patients who underwent colon surgery consecutively from the launch date to December 31, 2017, represented the study group. Exclusion criteria included blunt and penetrating trauma patients and those presenting with colon perforation and class IV wounds.

Our study defines colon surgeries as any operation involving the colon from the cecum to the rectum. Data for colon operations were determined according to Medicare CPT codes: 44,140–44,160, 44,204–44,213, 44,188, 44,206, 44,208, 44,320, 50810,57307, 44,346, 45,110–45,123, 45,395, and 45,397. We excluded isolated rectal and anal operations. Surgeons performing colon operations included general surgeons, colorectal surgeons, gynecological oncologists, and acute care surgeons.

### Definition of surgical site infection

We used the National Healthcare Safety Network criteria for reportable colon surgical site infections. Colon SSIs includes deep incisional and organ space infections occurring within 30 days after the procedure and meeting one of the following criteria:

- Purulent drainage from a drain placed into the organ space.
- Organisms identified from fluid or tissue in the area obtained by microbial testing.
- An abscess or infection detected on gross anatomical exam or imaging.
- Surgeon diagnosis.

We excluded superficial surgical site infections from the analysis.

### Colon bundle oversight team

We assembled a multidisciplinary colon bundle oversight team to oversee the quality initiative, consisting of surgeons, anesthesiologists, peri-operative nurses, floor nurses, infection prevention specialists, and quality improvement specialists. The team developed the data-driven bundle components, educated the stakeholders, and launched the bundle. The team collected and analyzed data to identify continuous improvement opportunities and provide real-time feedback to surgeons, nurses, and anesthesiologists. The group initially met weekly for the first month of implementation and then monthly.

### Colon SSI Reduction Bundle

**Fig. 1:**
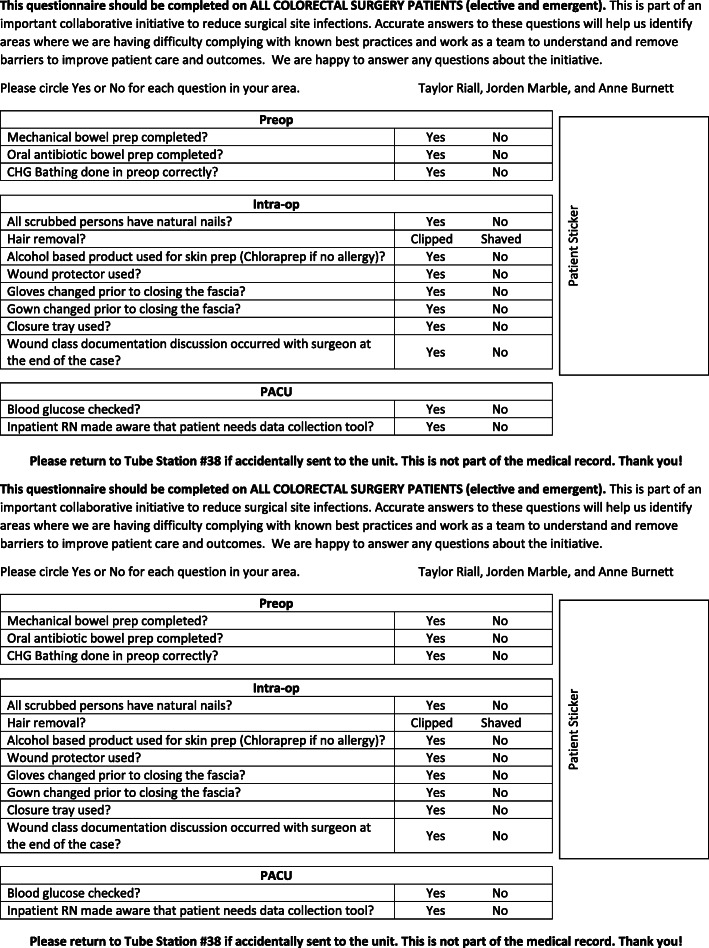
Colon SSI Bundle Checklist

The Colon Bundle became effective January 1, 2017. The bundle was standardized across the continuum of care including the preoperative, intraoperative, and immediate postoperative periods. Documentation of the bundle was initiated in the preoperative ready room (PRR). Nurses at each phase of care were charged with completing their respective portions of the bundle form (Fig. 1).

### Preoperative phase

#### Day before surgery

Patients undergoing elective surgery received oral antibiotics (neomycin and flagyl) the day before surgery (Table 1). The combination of preoperative oral antibiotics and prophylactic IV antibiotics was chosen because this combination has been shown to have a lower SSI rates than IV antibiotics alone [7, 8]. Mechanical bowel prep was not standardized for all surgeons and the decision to utilize bowel prep was left to the discretion of the surgeon. Studies supported both decisions to utilize mechanical bowel preparation along with oral antibiotics [9, 10] and oral antibiotics alone [8]. While mechanical bowel preparation was not required, it was tracked (Fig. 1). Both oral antibiotics and mechanical bowel prep were not utilized in emergent operations.

**Table 1:** Preoperative Bowel Prep

Preoperative Bowel Prep
Neomycin 1 gm PO and Metronidazole 500 mg PO Give both at 2 pm, 3 pm, and 10 pm the night before surgery
Mechanical bowel prep per attending preference

#### Day of surgery

Upon entry into the preoperative ready room (PRR), patients bathed with chlorhexidine gluconate (CHG) wipes and then changed into 3 M™ Bair Paws™ surgical gowns. Patient warming was initiated by heating the surgical gowns and infusing warm saline. Prophylactic intravenous (IV) antibiotics were administered within 60 min prior to skin incision. Antibiotic choices were chosen based on the current literature (13, 14). We chose the combination of cefazolin (2 grams, 3 gram is weight > 120 kg) and metronidazole (1000 mg) which has been shown to be significantly associated with lower SSI rates compared to cefoxitin. Studies have also demonstrated effectiveness of ertapenem relative to cefotetan and cefoxitin. There was significant debate about the use of ertapenem as this does not require redosing. However, ertapenem has been associated with increased rates of *Clostridium difficile* infection and has been associated with antibiotic resistance, leading to our choice of cefazolin and metronidazole. Patients who were allergic to penicillin or cephalosporins received Ciprofloxacin 400 mg and Metronidazole 1000 mg (Table 2). Antibiotic choice was confirmed in PRR but given in the operating room to ensure that antibiotics were given within 30 min of skin incision.

**Table 2:** Prophylactic Antibiotics Protocol

Prophylactic Antibiotics
Cefazolin 2 gm (3 gm if > 120kg) and Metronidazole 1000 mg IV - With Penicillin allergy: Ciprofloxacin 400 mg and Metronidazole 1000 mg IV
Intraoperative Antibiotic Redosing
Cefazolin every 4 hours Metronidazole if case > 8 hours - Dosing should be adjusted based on renal function. Cefazolin is renally eliminated - Prophylactic antibiotics are stopped at 24 hours

### Intraoperative phase

The intraoperative bundle focused on 3 areas: pre-incision, post-incision, and closing.

#### Pre-Incision

Patient warming was maximized by maintaining operating room temperatures above 70 degrees Celsius and applying under body warmers, warm IV fluid, and Bair Paws™. Patient core body temperatures were measured by temperature probe urinary catheters. Antibiotic administration was verified in the operating room and IV antibiotics were administrated in the operating room. The abdominal surgical sites were prepared by removing body hair with clippers and then prepping the skin with chlorhexidine and alcohol-based products. Skin was left to air-dry for 3 min prior to sterilely draping. Surgical personnel scrubbing into the case were required to have natural nails and underwent standard hand sterilization technique prior to gowning.

#### Post‐incision

The case proceeded as directed by the operating surgeon. Instruments and surgical technique were left to the discretion of the surgeon and not controlled. Several factors were standardized according to protocol. Dual-ringed wound protectors were utilized on all open cases. During laparoscopic cases, wound protectors were placed at the incision site utilized for colon externalization. Antibiotic redosing proceeded according to antibiotic type (Table 2). Patients were kept warm with Bair Hugger™, under body warmer, and warm IV fluid during the course of the procedure. Intraoperative irrigation was not standardized. A closing protocol was initiated after completion of the anastomosis or diversion and prior to closing fascia.

#### Closing

**Fig. 2:**
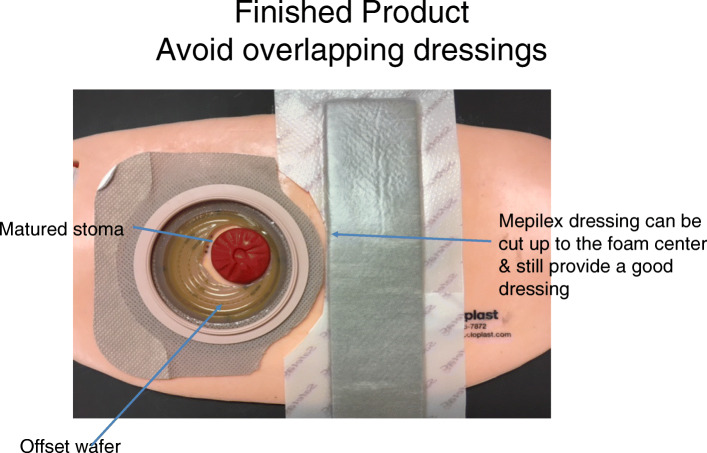
Wound Application

The closing protocol required that all surgical team members scrubbed during case change gowns and gloves prior to closing fascia. A new closing tray was opened on a new table and these fresh instruments were utilized for fascia and wound closure. Intraoperative dressings were placed in sequence – ostomy appliance (when indicated), followed by midline wound (Fig. 2). Skin was closed with staples in open cases and subcuticular sutures and Dermabond in laparoscopic cases. For contaminated wounds, the decision to leave a wound open was left to the discretion of the surgeon. The preferred midline dressing was the Mepilex® Border Ag dressing.

### Postoperative phase

The postoperative bundle components focused on maintaining euglycemia and proper wound coverage. The goal for glucose control was < 180 mg/dL for 48 consecutive hours. Glucose levels were checked on all patients (diabetic or non-diabetic) on arrival to the post-anesthesia care unit (PACU). A blood glucose level > 150 mg/dL triggered another glucose check 1 h later. All patients with blood glucose level > 150 mg/dL after two sequential checks were started on an insulin drip protocol. Diabetic patients with controlled blood glucose in the PACU and not requiring insulin drips were monitored regular glucose checks and sliding scale protocols according to physician preference. Wound dressings were not manipulated for at least 48-hours. Silver dressings were left in place for five days unless there was indication to remove the dressing and evaluate the wound. Bundle documentation was continued on the hospital units. Insulin drips did not require ICU care and were monitored on the floor with a protocolized order set.

### Data analysis

The primary endpoint of bundle effectiveness was overall colon surgical site infections, and the secondary endpoint was CMS reportable SSIs. We grouped data into two categories according to bundle implementation: Pre-bundle and Post-bundle. We used chi-square test or independent sample t-test to evaluate differences between groups.

We grouped surgical site infections into non-reportable (superficial SSIs) and reportable infections. Differences in reportable SSIs were compared between the two groups using a chi-square test. Statistically significant results were set to a *p*-value < 0.05 at an alpha of 0.05. For the entire period of the study, surgical site infection SIR targets were calculated by the NHSN through a logistic regression model that converts log-odds into a probability or risk of infection for each procedure by adjusting for the sum of risk factors (gender, age, BMI, ASA, closure technique, oncologic hospital). We evaluated our SIR before and after bundle implementation.

## Results

There were 277 patients in the pre-bundle group and 249 patients in the post-bundle group. Though women were predominant in both groups, there were no gender differences between the groups (Table 3). There was also no difference in mean age or mean BMI between both groups. Mean operating time did not vary between both groups. More than 88 % of cases in both groups were elective with no statistical difference between the groups.

**Table 3:** Patient Characteristics

	2016	2017	*p*-value
Patients, N	277	249	
Female, N (%)	149 (53.4 %)	136 (54.6 %)	0.85
Mean age, years (Std Dev)	57.9 (20.5)	56.7 (21.1)	0.46
BMI, N (%)			0.75
< 25	121 (43.7 %)	109 (43.8 %)	
25.0–29.9	77 (27.8 %)	60 (24.1 %)	
30.0–34.9	36 (13.0 %)	50 (20.1 %)	
35.0–39.9	25 (9.0 %)	20 (8.0 %)	
> 40	18 (6.5 %)	10 (4.0 %)	
ASA Class, N (%)			0.88
Class 1	7 (2.5 %)	10 (4.0 %)	
Class 2	94 (33.9 %)	90 (36.1 %)	
Class 3	141 (50.9 %)	109 43.8 %)	
Class 4	28 (10.1 %)	32 (12.9 %)	
Class 5	7 (2.5 %)	8 (3.2 %)	
Mean OR Time, Hr (Std Dev)	2.3	2.2	0.49
Emergent, N (%)	32 (11.6 %)	27 (10.8 %)	0.79

**Fig. 3:**
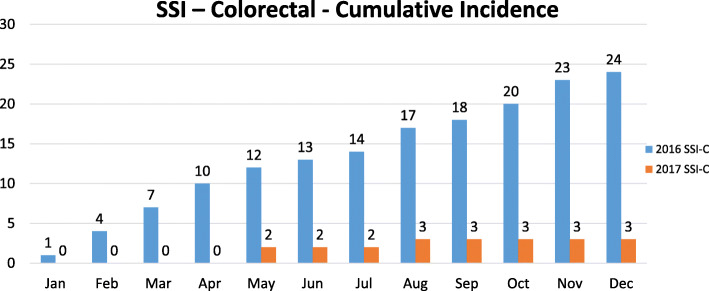
Colon Surgical Site Infections (Cumulative per Month)

Prior to implementing the bundle, the unadjusted SSI rate in 2016 was 8.7 % (24 SSIs in 277 patients, Table 4). At this SSI rate, the standardized infection ratio (SIR) of 3.08 was 3.7-fold higher than the expected target SIR for our hospital of 0.83. At the end of the first year after bundle implementation, the overall unadjusted SSI rate fell by 86.1–1.2 % (3 SSIs in 249 patients, Fig. 3). The decreased SSI rate from 2016 to 2017 was statistically significant (8.7 % vs. 1.2 %, *p* < 0.0001). The SIR fell by 85.4 % in 2017 to 0.45. To assure that the drop in the SSI rate did not result from factors other than the bundle, the raw SSI rates in 2015 and 2018 were evaluated (but not included in the data analysis). With comparable volume, there were a total of 22 colon SSI cases in 2015 (similar to the 24 reportable SSIs in 2016) and 4 in 2018 (comparable to the 3 in 2017), demonstrating sustained results.

**Table 4:** CMS Reportable Surgical Site Infection

	2016	2017
Patients, N	277	249
Total SSI	24	3
Raw SSI Rate (%)	8.7 %	1.2 %
Standardized infection ratio (SIR)	3.08	0.45
NHSN Target SIR	0.83	0.83

## Discussion

By 2016, our hospital was struggling with numerous colon surgical site infections. According to NHSN, our hospitals’ standardized infection rate exceeded the national average, nearly 4-fold. We did not isolate the problem to a few surgeons but instead determined it was a hospital issue. To improve colon surgery outcomes and optimize patient safety, we standardized our practice and developed a colon surgical site infection reduction bundle.

The colon bundle initiative launched on January 1, 2017. By December 31, 2017, our hospital had achieved an 86.1 % reduction in colon SSI rates and an 85.4 % reduction in the national standardized infection rate. With comparable operative volume, we succeeded in reducing our annual colon surgical site infections from 24 to 2016 to 3 in 2017.

Prior studies implementing Colon Bundles have shown a significant reduction in superficial SSI rates from 57 to 70 %, but mixed effects on deep SSI rates [4, 5]. Though some studies show a 70 % reduction in deep SSI rates [9], others show no effect [4]. Our Colon Bundle implementation shows that meaningful reduction in deep organ space infections is achievable. Standardizing our patients’ care in all three perioperative phases helped achieve an 86 % reduction in deep and organ space infections and a standardized infection ratio reduction 46 % below the national target. We also demonstrate that a colon bundle sustains outcomes by reporting only four colon SSIs in 2018 (data not shown).

Several other reasons could account for the reduction in colon SSIs. For one, individual surgeon factors can contribute to patient outcomes. However, between 2016 and 2018, we did not have any surgeon turnover or acquisition that could have impacted the data. Another plausible explanation for the reduction could be shifting procedures from generalists to colon specialists. However, the volume across specialties remained stable, and our colon surgeons did not incur an increase in volume during this period. Another possibility that accounts for our divergence in outcomes before and after our colon initiative is standardization. Before launching our colon bundle, each surgeon operated on and managed their patient according to individual preference. After launching our initiative, all of our surgeons followed a data-driven protocol. Even the mundane practice of changing surgical gloves after creating the anastomosis was standardized. We infer that standardizing our practices contributed to our success.

Our study has some limitations. The most glaring limitation is that this is not a prospective randomized controlled study. Secondly, the data presented is only for two calendar years. However, the four colon SSIs in 2018 show that our approach is sustainable and not limited to a single year. Another limitation is that we do not account for patient comorbidities. Though we acknowledge that comorbidities may impact SSIs, we are a tertiary referral center, and our patient severity remains stable year-over-year and feel confident that this is not affecting our outcomes.

We demonstrate that incorporating a colon bundle can reduce surgical site infections and improve quality outcomes and patient safety. We recommend standardizing all three phases of perioperative patient care in all patients undergoing elective or emergent colon surgery.

## Conclusions

Implementing a standardized surgical site infection bundle can successfully reduce surgical site infections in colon surgery. We recommend standardizing all three phases of perioperative patient care in colon surgery through a rigorous and data-driven colon bundle.

## Data Availability

Non-patient Data will be made available as needed.

## References

[1] Shaw E, Gomila A, Piriz M, Perez R, Cuquet J, Vazquez A (2018). Multistate modelling to estimate excess length of stay and risk of death associated with organ/space infection after elective colorectal surgery. J Hosp Infect.

[2] Kobayashi M, Mohri Y, Inoue Y, Okita Y, Miki C, Kusunoki M (2008). Continuous follow-up of surgical site infections for 30 days after colorectal surgery. World J Surg.

[3] Bhakta A, Tafen M, Glotzer O, Ata A, Chismark AD, Valerian BT (2016). Increased Incidence of Surgical Site Infection in IBD Patients. Dis Colon Rectum.

[4] Turner MC, Migaly J (2019). Surgical Site Infection: The Clinical and Economic Impact. Clin Colon Rectal Surg.

[5] Keenan JE, Speicher PJ, Thacker JK, Walter M, Kuchibhatla M, Mantyh CR (2014). The Preventive Surgical Site Infection Bundle in Colorectal Surgery an Effective Approach to Surgical Site Infection Reduction and Health Care Cost Savings. JAMA Surg.

[6] Hoang SC, Klipfel AA, Roth LA, Vrees M, Schechter S, Shah N (2019). Colon and rectal surgery surgical site infection reduction bundle: To improve is to change. Am J Surg.

[7] Lutfiyya W, Parsons D, Breen J. A colorectal “care bundle” to reduce surgical site infections in colorectal surgeries: a single-center experience. Perm J. 2012 Summer;16(3):10–6. 10.7812/tpp/12.968 PMC3442755 23012593

[8] Ghuman A, Chan T, Karimuddin AA, Brown CJ, Raval MJ, Phang PT (2015). Surgical Site Infection Rates Following Implementation of a. Dis Colon Rectum.

[9] Deierhoi RJ, Dawes LG, Vick C, Itani KM, Hawn MT (2013). Choice of intravenous antibiotic prophylaxis for colorectal surgery does matter. JAMA Surg.

[10] Scarborough JE, Mantyh CR, Sun Z, Migaly J. Combined Mechanical and Oral Antibiotic Bowel Preparation Reduces Incisional Surgical Site Infection and Anastomotic Leak Rates After Elective Colorectal Resection: An Analysis of Colectomy-Targeted ACS NSQIP. Ann Surg. 2015;262(2):331-7. 10.1097/SLA.0000000000001041 26083870

